# Sickness absence and disability pension before and after first childbirth and in nulliparous women by numerical gender segregation of occupations: A Swedish population-based longitudinal cohort study

**DOI:** 10.1371/journal.pone.0226198

**Published:** 2019-12-13

**Authors:** Krisztina D. László, Charlotte Björkenstam, Pia Svedberg, Petra Lindfors, Kristina Alexanderson

**Affiliations:** 1 Division of Insurance Medicine, Department of Clinical Neuroscience, Karolinska Institutet, Stockholm, Sweden; 2 Department of Public Health Sciences, Karolinska Institutet, Stockholm, Sweden; 3 Department of Psychology, Stockholm University, Stockholm, Sweden; University of Alicante, SPAIN

## Abstract

**Background:**

Pregnancy is associated with a temporarily increased sickness absence (SA) risk. This association may vary by the level of occupational gender segregation; however, knowledge in this area is limited. We studied whether trends in SA and disability pension (DP) in the years before and after first childbirth among women with one or more childbirths and with no childbirth during the study period varied by occupational gender segregation.

**Methods:**

We conducted a population-based register study involving nulliparous women aged 18–39 years, living in Sweden in 2002–2004 (n = 364,411). We classified participants in three childbirth groups as: (1) no childbirth in 2005 or in the next 3.75 years, (2) first childbirth in 2005 and no births in the subsequent 3.75 years, and (3) first childbirth in 2005 and at least one additional birth in the subsequent 3.75 years, and into five categories based on the rate of women in their occupations. We compared crude and standardized mean annual net SA and DP days during the three years before and the three years after 2005 across the childbirth and occupational gender segregation categories.

**Results:**

Women in extremely male-dominated occupations had or tended to have somewhat higher mean combined SA and DP days than women in gender-integrated occupations; women in female-dominated occupations had comparable or tended to have slightly higher mean SA and DP days than women in gender-integrated occupations. Except for the year before the first childbirth, women who gave birth, especially those who gave several births, had generally a lower mean combined standardized SA and DP days than nulliparous women. We found no substantial differences in trends in SA and DP around the time of first childbirth according to occupational gender segregation.

**Conclusions:**

Trends in SA and DP around the time of first childbirth did not vary by occupational gender segregation.

## Introduction

It is well-established that employed women have on average higher levels of sickness absence (SA) than men [1]. Childbirth has been suggested to contribute to the explanation of these differences. Pregnancy and childbirth are associated with a temporarily higher risk of SA [2–7], with the majority of women having at least one SA spell at some point during pregnancy [8–10]. In a recent study, we have also found that women who gave birth during the study period had higher mean combined SA and disability pension (DP) days during the year before their first delivery than their counterparts who remained nulliparous; otherwise women who gave birth, especially those with several births, had fewer days of combined SA and DP than women not giving birth [11].

Several studies suggest that the higher SA level during pregnancy may not be fully attributable to pregnancy-related medical conditions and emphasize a need to identify non-medical factors that may contribute to the higher levels [9, 12]. Women’s experiences of pregnancy, their perceptions about job demands, and their attitudes to SA during pregnancy may be sensitive to the culture and the norms of their occupation [13, 14]. An increasing number of studies document an association between the numerical gender composition, i.e., the proportion of women and men, in an occupation and SA [15–22]. Based on Kanter’s theory of tokenism [15, 23], gender-minority groups in an occupation have been hypothesized to have higher SA than the majority due to the minority position being associated with higher stress and lower social resources, and subsequently poorer health and lower motivation to go to work [15, 23]. Furthermore, high physical demands and that work routines primarily fit men may also increase SA among pregnant women working in male-dominated occupations. Yet, some absence culture theories hypothesize that female-dominated occupations have more lenient norms regarding reasons and acceptable levels of work absence, which in turn may result in higher SA levels than in gender-integrated occupations [15, 24]. Several female-dominated occupations may allow more flexibility to combine work and family life than other occupations, which may involve a selection of some absence-prone women in these occupations [14]. Furthermore, as several extremely male- and extremely female-dominated occupations require low skill levels, women with low education and with health problems are more likely to be selected in these occupations [14]. Low socioeconomic status and preexisting morbidity increase the risk of pregnancy complications [25–29], which may further contribute to higher SA during pregnancy in women in gender segregated than in gender-integrated occupations.

In line with some of the above hypotheses, a few studies have found a U-shaped or an inverse J-shaped association between the proportion of women in an occupation and SA [15–20]. Only two studies have investigated the associations between the gender segregation of occupations or workplaces, childbirth and SA. Alexanderson et al. [19] found a U-shaped association between occupational gender segregation and SA due to pregnancy-related diagnoses (i.e., abortion, preeclampsia, bleeding, urinary infections, early labor, backache, and fatigue due to pregnancy) among all pregnant women in a Swedish county. Melsom [14] reported a positive association between the proportion of women at the workplace and the number of SA days during pregnancy. Both studies had a cross-sectional design, i.e. assessed occupational or workplace gender segregation and SA only during pregnancy and none had a comparison group of non-pregnant women, which limited possibilities to consider health selection into pregnancy. None of the studies investigated DP. Furthermore, given substantial changes in maternal age and health at first childbirth, in work organization and in the labour market, as well as in regulations concerning SA and pregnancy-related social benefits since 1985 –when the study of Alexanderson et al. [19] was conducted–a further study in this area with more recent data is needed.

We studied whether trends in SA and DP during the years before and after first childbirth among women with one or more childbirths, and among women with no childbirth during the study period, varied according to the level of their occupational gender segregation. By analyzing SA/DP three years before the childbirth and two groups of women with and one group of women with no childbirth, we aimed to obtain indications of the importance of health selection into childbirth.

## Materials and methods

### Study population and design

This is a longitudinal population-based cohort study based on anonymized microdata linked from several nationwide Swedish registers. Linkage was possible through the unique personal identification number assigned to all residents in Sweden [30]. The study population consisted of all nulliparous women aged 18–39 years registered as living in Sweden during 2002–2004; we used information on residency from the Longitudinal Integration Database for Health Insurance and Labor Market Studies (LISA) and data on previous births from the Medical Birth Register (MBR) and the National Patient Register. The LISA database, held by Statistics Sweden, includes extensive information on occupation and several other sociodemographic factors for all individuals residing in Sweden at the end of each calendar year. The other three used registers are held by the National Board of Health and Welfare. The MBR, established in 1973, covers 97–99% of the births in Sweden and includes information on date of delivery, parity, pregnancy-related characteristics, and pregnancy outcomes [31]. In order to increase coverage on childbirths, we searched the National Patient Register to obtain information on births not found in the MBR [32]. Hospitalizations with a main or a secondary diagnosis related to childbirth (as defined by the International Classification of Disease (ICD) codes: ICD-7: 660, 670–678; ICD-8: 650–662; ICD-9: 650, 651, 652, 659X,W/659.W-659.X, 669.E,F,G,H,W,X; ICD-10:O75.7-O75.9, O80-84) were obtained from the Patient Register, established in 1964 and nationwide since 1987. If a childbirth appeared in both registers, we used the date from the MBR. Information on death of study participants after 2005 was obtained from the Cause of Death Register.

To avoid that the outcome (SA/DP) was influenced by a new pregnancy, women in the three exposure groups having a first childbirth in the 43 weeks after Y_+3_ (the third follow-up year) were excluded. Of the identified 492,504 nulliparous women aged 18–39 years living in Sweden in 2001–2004, those 364,411 with information on occupation in 2004 or in 2005 were included in the analyses for the present study.

The project was approved by the Regional Ethical Review Board of Stockholm.

### Measures

#### Exposure: Childbirths

Women were classified based on their childbirth status as: (1) no birth before or in 2005, nor in the next three years and 43 weeks, i.e. approximately 9 months (group “B0”), (2) giving birth to a first child in 2005 with no additional birth in the next three years and 43 weeks (group “B1”), and (3) giving birth to a first child in 2005 with at least one additional birth in the next three years (group “B1+”). The study period ranged from the three years before to the three years after the index date, that is, Y_-3_ through Y_+3_. The index date (T_0_) was defined as the date of the first childbirth for groups B1 and B1+, while for group B0 T_0_ was set to 2 July 2005. In order to avoid that a later pregnancy affected SA during the three years after T_0_, all women were followed for an additional 43-week period, i.e., approximately 9 months, after year 3, to consider any pregnancy that started in Y_+3_. Thus, if a women giving birth in 2005, also did that in the period up through Y_+3_ + 43 weeks, she was classified as belonging to the B1+ group.

#### Outcomes: SA and DP

Information on start and end dates of SA and DP during the study period was obtained from the MIDAS (Swedish acronym for Microdata for Analysis of the Social Insurance) database held by the Swedish Social Insurance Agency [33]. Individuals aged 16 and above, living in Sweden, and with income from work or unemployment benefits can receive SA benefits if their work capacity is reduced due to disease or injury [34]. For employed individuals, the employer generally pays for the first 14 days of a SA spell, after which the Swedish Social Insurance Agency pays. Here we used information on SA spells >14 days. All in Sweden aged 19–64 who have long-term or permanent work incapacity due to disease or injury can be granted DP. The SA benefits cover 80% of the lost income, up to a certain limit, while DP covers up to 65%. Both SA and DP can be granted full- or part-time (25%, 50%, 75%) of ordinary working hours. Part-time SA and DP was transformed into net days, e.g., two absence days on 50% were regarded as one net day. The mean combined SA and DP annual net days was the main outcome; we analyzed (1) mean annual net SA days, (2) mean annual net DP days, and (3) having at least one SA or DP spell per year as additional outcomes.

#### Effect modifier: Five occupational gender segregation categories

We retrieved information on occupation in 2004 from LISA. If information on occupation in 2004 was missing, we used data on occupation from 2005, if available. Occupation was coded according to the Swedish Standard Classification of Occupations 2012 [35]. We categorized occupation based on their three-digit Swedish Standard Classification of Occupations codes as (1) extremely male-dominated (if the proportion of men in the occupation in LISA 2004 was >90%), (2) male-dominated (>60% and ≤90% men), (3) gender-integrated (40–60% men/women), (4) female-dominated (>60% and ≤90% women), and (5) extremely female-dominated (>90% women).

#### Confounders: Sociodemographic factors

The following sociodemographic factors were obtained from LISA (December 2004): (1) age (categorized as 18–24, 25–29, 30–34, and 35–39 years), (2) country of birth (categorized as Sweden, Other Norther Europe, Other EU25 versus rest of the world), (3) living area (classified based on the H-classification scheme [36]) as a large city (Stockholm, Gothenburg, Malmö), a medium-sized city (≥90,000 inhabitants), and a small city/village (<90,000 inhabitants)), (4) educational attainment (categorized as elementary (≤9 years), high school (10–12 years), and university/college (≥13 years), and (5) family situation (married or in registered partnership versus single).

### Statistical analyses

All analyses were stratified according to occupational gender segregation. For each of the three childbirth groups, crude and standardized annual mean net SA days, DP days, and their combination were calculated in the three years before and in the three years after T_0_. To consider confounders of the association between childbirth and SA/DP, we standardized the mean annual net SA days, DP days, and their combination by age, country of birth, type of living area, and educational level in 2004. We performed direct standardization using the PROC MIXED procedure in SAS, with the B1 group as the reference. Potential confounders were chosen based on our a-priori hypothesis regarding the most important differences in our three childbirth groups and based on the previous literature about factors that predict SA/DP. In addition, we also calculated the percentage of women with at least one SA/DP spell per year during each of the three years before and the three years after T_0_.

Analyses were conducted using SAS 9.4.

## Results

Of the 364,411 women included in the study, 1.5% worked in extremely male-dominated occupations, 14% in male-dominated occupations, 10.6% in gender-integrated occupations, 69.2% in female-dominated occupations, and 4.8% in extremely female-dominated occupations; 90.3% had no birth before or during the study period (B0), 3.4% had their first childbirth in 2005 and no additional births during follow-up (B1), while 6.3% had their first childbirth in 2005 and at least another birth during the follow-up (B1+). Table 1 shows the distribution of age, country of birth, type of living area, educational level, and family situation by occupational gender segregation and childbirth status. The proportion of women who gave at least one birth during the study period (i.e. in B1 or B1+) was highest in the extremely female-dominated group and lowest in the extremely male-dominated and in the female-dominated groups (S1 Table).

**Table 1:** Cohort characteristics by occupational gender composition and childbirth (N = 364,411).

Factors	Groups by occupational gender composition and childbirth														
	Extremely male-dominated (n = 5,363) %			Male-dominated (n = 50,938) %			Gender-integrated (n = 38,692) %			Female-dominated (n = 252,041) %			Extremely female-dominated (n = 17,371) %		
	B0	B1	B1+	B0	B1	B1+	B0	B1	B1+	B0	B1	B1+	B0	B1	B1+
**Total %**	92.5	2.7	4.8	87.4	4.2	8.4	86.4	4.3	9.3	92.0	3.0	5.0	81.3	5.5	13.2
**Age**															
**18–24**	43.7	23.3	26.3	33.2	9.5	9.5	27.2	5.6	5.6	58.8	28.8	26.8	21.9	5.3	8.7
**25–29**	23.8	33.6	35.3	28.3	30.3	39.7	30.1	27.9	37.7	21.5	36.1	44.9	31.2	42.1	54.5
**30–34**	15.7	27.4	29.0	20.5	38.3	42.0	23.6	44.1	47.6	11.0	24.9	24.3	24.3	35.9	31.9
**35–39**	16.8	15.8	9.4	18.0	21.9	8.8	19.1	22.5	9.2	8.7	10.2	4.0	22.6	16.7	4.8
**Country of birth**															
**Sweden**	92.7	91.8	97.3	90.6	90.0	93.8	88.8	89.7	93.8	89.3	87.6	91.8	90.4	91.3	94.4
**Other Northern Europe**	1.3	0	0	1.4	1.9	1.2	1.5	1.9	1.2	0.9	1.2	0.8	2.1	1.9	1.4
**Other EU25**	1.1	0.7	0.4	1.5	2.0	1.3	2.3	2.2	1.1	1.2	1.1	0.8	1.3	1.3	0.9
**Rest of the world**	5.0	7.5	2.4	6.6	6.6	3.6	7.4	6.2	3.9	8.7	10.2	6.7	6.1	5.6	3.3
**Type of living area**															
**Large city**	36.6	40.4	32.2	48.8	50.8	53.5	52.3	56.5	58.2	42.8	40.2	38.6	47.1	42.4	40.0
**Medium-sized city**	38.5	32.9	41.2	32.9	29.7	29.3	32.4	29.3	28.6	36.1	35.2	36.2	35.5	34.1	38.9
**Small city/village**	25.0	26.7	26.7	18.2	19.4	17.1	15.3	14.2	13.2	21.0	24.6	25.3	17.4	23.5	21.0
**Educational attainment**															
**Elementary school (≤9 years)**	13.0	7.5	7.1	8.8	6.2	3.3	7.0	3.1	1.6	13.4	10.4	6.6	2.5	1.4	0.6
**High school (10–12 years)**	58.8	67.1	62.8	47.3	45.9	36.7	31.7	25.5	17.4	53.6	58.4	50.6	21.0	15.2	9.2
**University/college (≥13 years)**	28.2	25.3	30.2	43.9	48.0	60.0	61.3	71.4	81.0	33.0	31.2	42.8	76.5	83.5	90.2
**Family situation**															
**Married or registered partnership**	5.0	13.7	14.5	6.6	26.5	31.8	7.2	31.5	37.2	4.2	19.7	24.0	8.4	27.2	30.4
**Single**	95.0	86.3	85.5	93.4	73.5	68.2	92.8	68.5	62.8	95.8	80.3	76.1	91.6	72.8	69.6

B0 = no childbirth before or in 2005, nor during the subsequent 3.75 years, B1 = first childbirth in 2005 and no births during the subsequent 3.75 years, B1+ = first childbirth in 2005 and at least one more birth during the subsequent 3.75 years.

**Fig 1:**
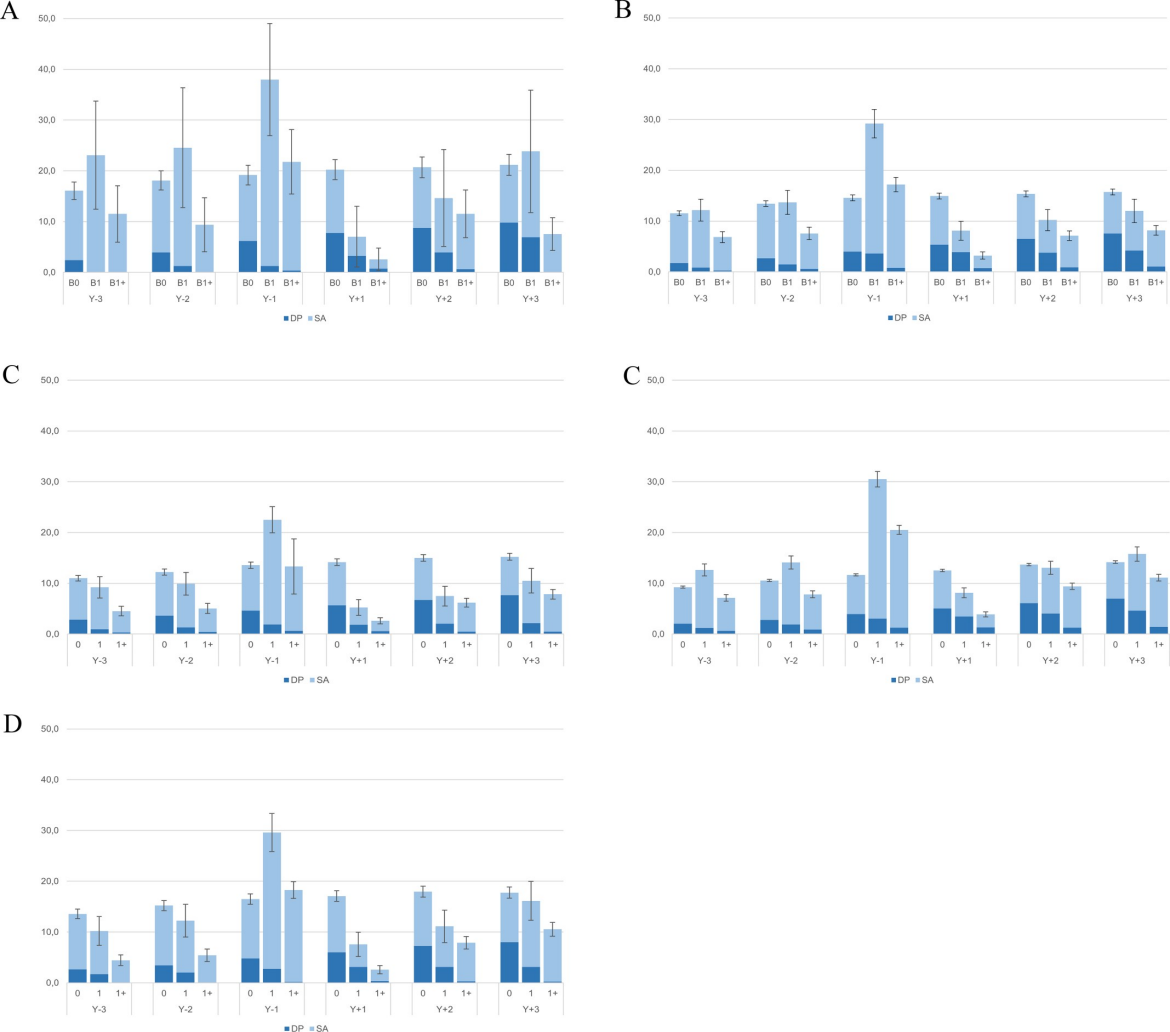
Crude mean sickness absence (SA) and disability pension (DP) net days/year and 95% confidence intervals within the (1a) extremely-male dominated, (1b) male-dominated, (1c) gender integrated, (1d) female-dominated, (1e) extremely female-dominated groups, by childbirth group and study year.

**Fig 2:**
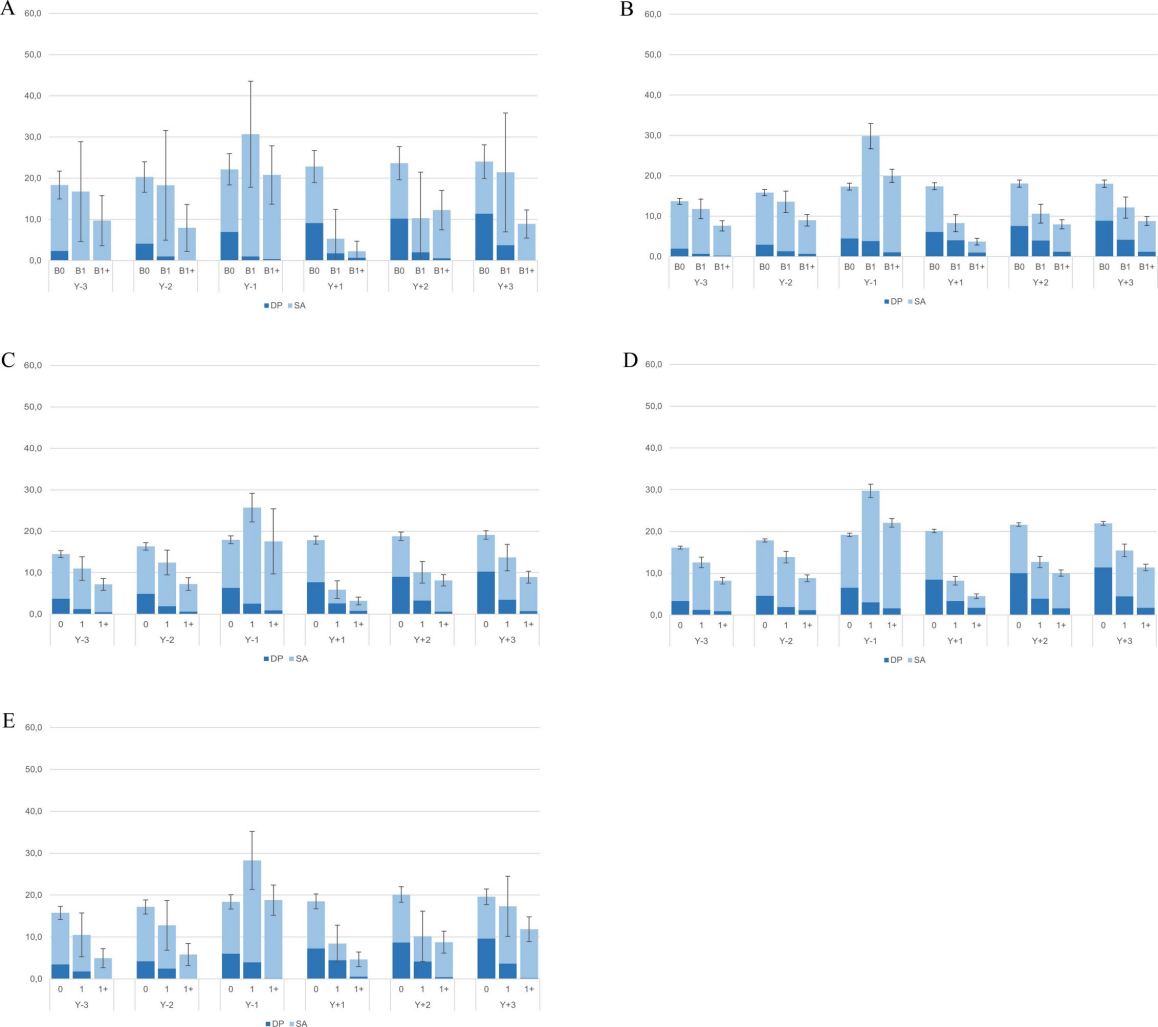
Standardized mean sickness absence (SA) and disability pension (DP) net days/year and 95% confidence intervals within the (1a) extremely male-dominated, (1b) male-dominated, (1c) gender-integrated, (1d) female-dominated, (1e) extremely female-dominated groups, by childbirth group and study year.

**Fig 3:**
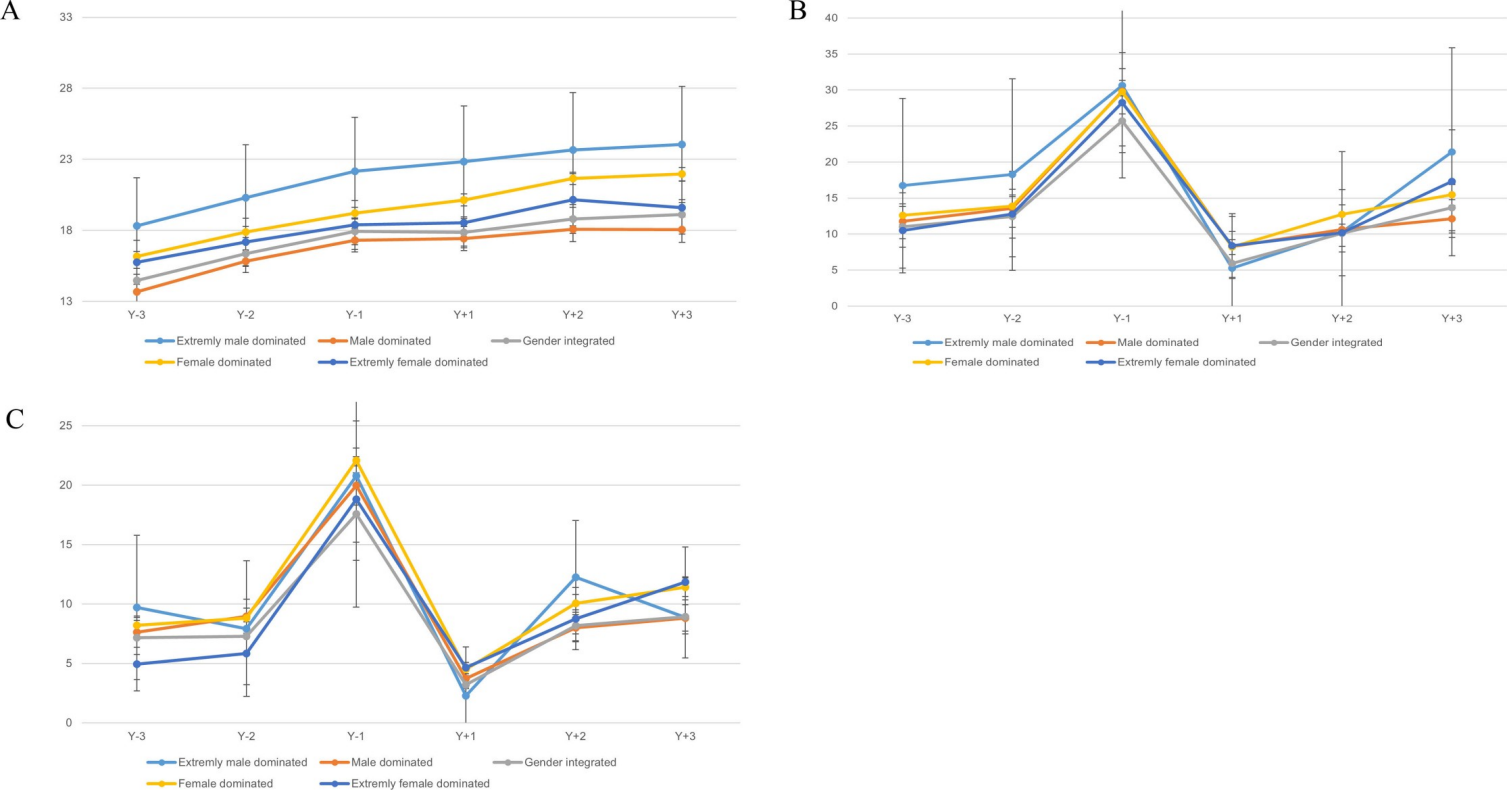
Standardized mean sickness absence (SA) and disability pension (DP) net days/year by occupational gender segregation category, and study year within each of the three childbirth groups (B0, B1, B1+).

Generally, women in the extremely male-dominated occupations had or tended to have the highest crude and standardized mean combined SA and DP days (Figs 1 and 2). Overall, women in female-dominated and in extremely female-dominated occupations tended to have slightly higher crude mean combined SA and DP days than those in gender-integrated occupations, but lower than those in the extremely male-dominated category. These differences attenuated in the standardized analyses. In most occupational gender segregation categories and during most study years, nulliparous women had or tended to have higher mean standardized combined SA and DP days than women giving birth, except for the year preceding the first childbirth in B1 and B1+. Generally, women with more than one childbirth had or tended to have fewer mean combined SA and DP days than those with one childbirth only. In all five occupational gender segregation categories, mean standardized combined SA and DP days of parous women were highest during the year before childbirth and lowest during the year after delivery. There were no substantial differences in trends in the number of net combined SA and DP days around the time of childbirth across occupational gender segregation categories; the confidence intervals corresponding to the mean standardized combined SA and DP days of women in B1 and B1+ in the gender integrated and in the gender segregated groups overlapped in case of several study years, including Y_-1_, suggesting that the differences among these groups were generally not statistically significant (Fig 3).

**Fig 4:**
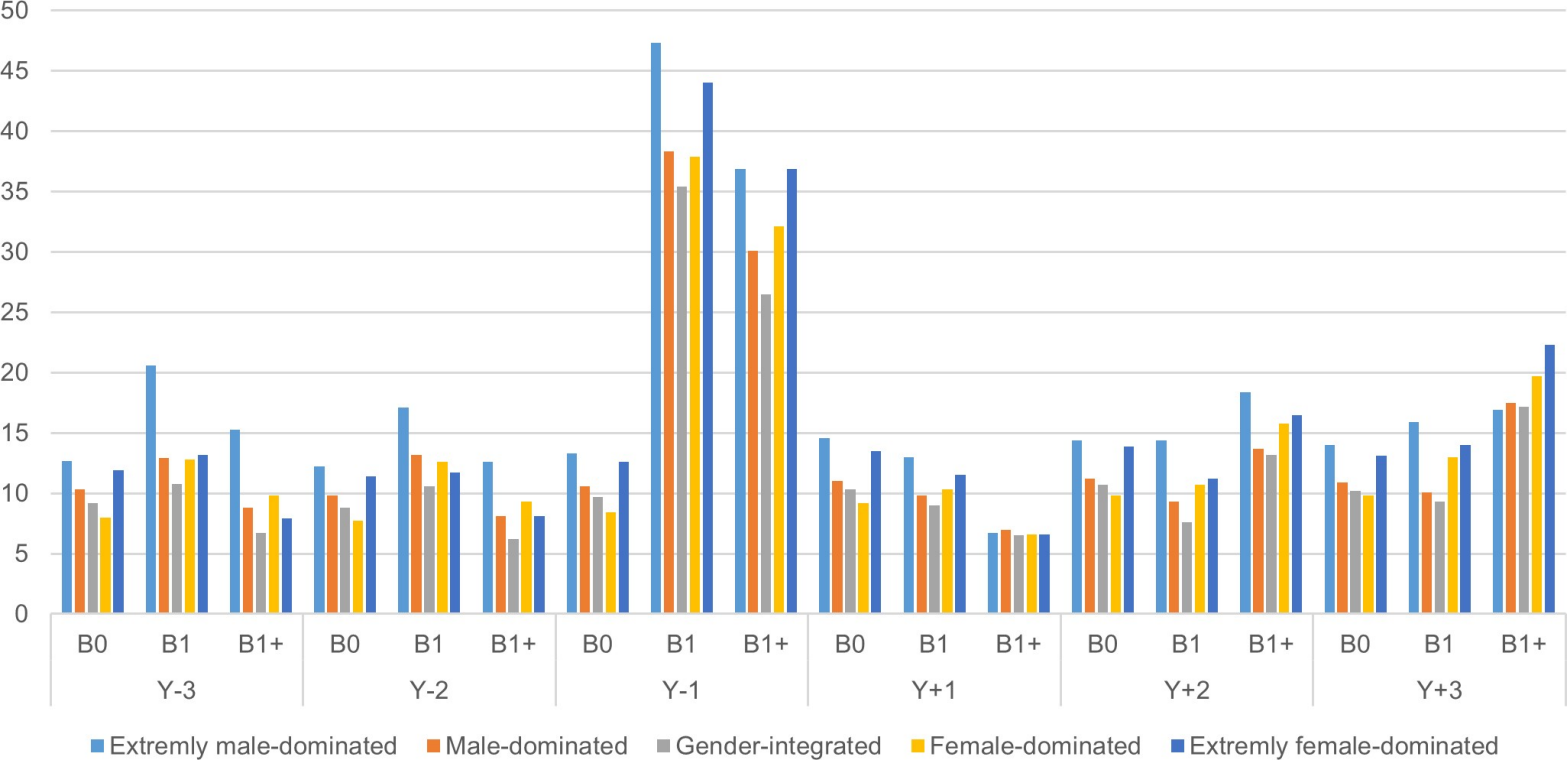
Proportion of women with any sickness absence spell >14 days and/or disability pension during the six study years, by gender segregation occupational category and childbirth group. B0 indicates no childbirth before or in 2005, nor during the subsequent 3.75 years, B1 having the first childbirth in 2005 and no births during the subsequent 3.75 years and B1+ having the first childbirth in 2005 and at least one more birth during the subsequent 3.75 years.

The associations between occupational gender segregation and having at least one SA or DP spell were largely similar to those from the main crude analyses with the continuous combined SA and DP outcome (Fig 4).

## Discussion

This study investigated whether trends in SA and DP in the three years before and the three years after the date of the first childbirth among women with one or more childbirths and among women with no childbirth during the study period varied according to the level of occupational gender segregation. We found that the combined net SA and DP days increased in the year before first childbirth. However, except for this year, women who gave birth, especially those who gave several births, had generally lower mean SA/DP days than women who remained nulliparous. The trends in SA and DP before and after the first birth did not differ substantially according to the level of occupational gender segregation.

The finding that women who gave birth during the follow-up–in particular those who gave several births–had lower combined SA and DP days two and three years before and after the first childbirth relative to their counterparts who remained nulliparous are in line with those of the few previous studies in this field [5, 6], including our recent study among all nulliparous women aged 18–39 years living in Sweden in 1994, 1999 and 2004 [11]. They suggest a health selection into childbirth, i.e. that women with poor health or other related characteristics are less likely to engage in pregnancy than their healthier counterparts [37, 38]; these differences persisted also after standardization for age, education, country of origin and marital status. Further studies with information on morbidity (e.g. specific medical diagnoses) need to more closely investigate the importance of health selection into giving birth [11]. A Swedish twin study, which by design could account for an important part of the genetic and environmental confounding factors shared by sisters, found evidence for a similar health selection into giving birth; an important finding of that study was that DP was often proceeded by several hospitalizations [39].

Similarly, our findings that the combined SA and DP days was higher the year before the date of first childbirth than in the other study years follow previous studies reporting an increase in SA levels during pregnancy [2–7, 11]. During pregnancy, many women experience different types of symptoms that can affect their work capacity and in some occupations it might be more difficult to adjust work conditions accordingly. In line with Kanter’s theory [15, 23] one could expect that it may be most difficult to alter work conditions in male-dominated occupations, where the work environment to a larger extent is structured with men as the norm. This, in addition to the negative psychosocial factors associated with a minority status, including possibly less positive attitudes towards symptoms of pregnancy, may result in higher SA during pregnancy in women in extremely male-dominated occupations than those in gender-integrated occupations. In line with several population-based studies we found generally higher combined SA and DP levels among women in extremely male-dominated than among gender-integrated occupations [15–19]. Similarly, in their study involving all pregnant women in a Swedish county Alexanderson et al. found a U-shaped association between occupational gender segregation and SA due to pregnancy-related diagnoses (i.e., abortion, preeclampsia, bleeding, urinary infections, early labor, backache, and fatigue due to pregnancy) [19]. Melsom [14], using another measure of SA, did not find this U-shape, rather a small positive association between the proportion of women at the workplace and the number of SA days during pregnancy. However, Melsom et al. [14] studied gender segregation of workplaces, we and Alexanderson et al. [19] of occupations; the two measures may differ as women can work in a female-dominated occupations (e.g., secretary) at a male-dominated workplace (e.g., metal industry) [15]. Our findings regarding no differences in the increase of SA and DP the year before childbirth among women working in gender segregated compared to those working in gender-integrated occupations, suggest that employers might have adjusted working conditions to the pregnancy situation, as required by the law. Job adjustments during pregnancy have been associated with a reduced risk of SA [9, 40]. Furthermore, women with physically strenuous and monotonous jobs allowing no adjustment of work conditions to pregnancy demands are entitled to “pregnancy benefits” in the 10–60 days prior to the expected delivery date. Moreover, all pregnant women, regardless of their employment status and work conditions, can make use of 60 days of the 480-day parental-leave benefit during the last two months of pregnancy [34]. These two benefits were not included in our analyses of SA and DP days.

The findings that women in female-dominated occupations had/tended to have a higher crude mean SA than women in gender-integrated occupations, with these differences being attenuated in the analyses standardized for age, education, country of origin and family situation, may also follow the hypothesis that women prone to absences are more likely to choose or to be selected into occupations dominated by women [14] which in turn seem to involve poorer work environments. However, many of the female-dominated occupations are physically strenuous, often more so than the male-dominated occupations, which is a strong risk factor for SA/DP [41], especially during pregnancy. We found that women in gender-integrated occupations were more likely to have university/college education and be married/in registered partnership than women in the female-dominated occupations, suggesting that the former category may have a healthier profile or more resources. If any small differences in SA/DP remained after standardization, these might be explained by residual confounding by health-related characteristics, differences in working conditions, or more lenient norms regarding SA among women in female-dominated occupations. Several occupations in women-dominated occupations may allow a looser connection to the labour market than the gender-integrated occupations, thus women with poor health may be more prone to select or to be selected into such occupations; unfortunately, we did not have information on these factors to explore their importance.

The strengths of this study include that all women in a country fulfilling our inclusion criteria could be studied, the large sample size, the longitudinal design, the nationwide high-quality register data that allowed us to include virtually all occupations, and the high quality information on childbirth and SA/DP [30, 32]. Also, the very high employment rate of women in Sweden reduces selection bias due to the healthy worker effect. Limitations are related to that although the Medical Birth Register covers between 97–99.5% of all births in Sweden from 1973, not all deliveries are included. We made a very strong effort to scrutinize the National Patient Register to identify all other childbirths back through 1964 for most of Sweden. For those identified through the National Patient Register we however, do not have information about parity, that is, they might have given birth in another country before moving to Sweden. This means that some of the women classified as not having given birth before 2005 might have done so. The inclusion criteria of having had to be living in Sweden 2001–2004 was one way to overcome this possible bias. Another aspect is that we did not have information about SA spells ≤14 days. This can be seen as both a strength and a limitation. It may be a strength as we only included the more long-term, serious SA spells, and a limitation as we did not have all SA days, resulting in an underestimation of annual SA days. However, short SA spells only account for a limited number of the total number of annual SA days [42]. Furthermore, though we tried to consider possible health selection into childbirth at the stage of the study design, i.e. (1) by including in the analyses all nulliparous women aged 18–39 years living in Sweden in December 2014 and not a sample, (2) three years before the childbirth and (3) standardizing our analyses by several sociodemographic factors, we cannot exclude the possibility of residual confounding, for example from previous morbidity. Similarly, women in different occupational gender segregation groups may differ in sociodemographic and health-related characteristics that we did not have the possibility to consider, which in turn may have contributed to differences in SA/DP among the groups. However, we did not find important differences in trends in SA/DP in the years around childbirth, which was the focus of our study. Also, our findings may only be generalized to countries with a welfare system providing healthcare and SA/DP benefits to all, with sociocultural contexts comparable to that of Sweden and with a high gender equality, high proportion of employed working women, and a strongly gender segregated labour market [43].

## Conclusions

In conclusion, SA and DP among women vary by the level of gender segregation of their occupations and by their childbirth status; combined net SA and DP were highest the year before first childbirth among women giving birth, but otherwise were generally lower among women giving birth than among those not giving birth, suggesting a health selection into pregnancy. These trends in SA and DP the years around first birth did not differ substantially according to the level of the occupational gender segregation.

## Supporting information

S1 Table Proportion of women with different childbirth status according to occupational gender segregation. (DOCX)
