# Supplementary material for: Sickness absence and disability pension before and after first childbirth and in nulliparous women by numerical gender segregation of occupations: A Swedish population-based longitudinal cohort study
Source: PLoS One. 2019 Dec 13;14(12):e0226198. doi: 10.1371/journal.pone.0226198 (PMC6910695; doi:10.1371/journal.pone.0226198)
Supplement: S1 Table — (DOCX) [file pone.0226198.s001.docx]

Supporting information

**S1 Table.** **Proportion of women with different childbirth status according to occupational gender segregation**

| **Occupational gender segregation group** | **Childbirth group** | | |
| --- | --- | --- | --- |
|  | **B0**  **N (%)** | **B1**  **N (%)** | **B1+**  **N (%)** |
| **Extremely male-dominated** | 4968 (92.53) | 146 (2.72) | 255 (4.75) |
| **Male-dominated** | 44511 (87.38) | 2162 (4.24) | 4265 (8.37) |
| **Gender-integrated** | 33413 (86.36) | 1666 (4.31) | 3613 (9.34) |
| **Female-dominated** | 231885 (92.0) | 7569 (3.00) | 12587 (4.99) |
| **Extremely female-dominated** | 14120 (81.28) | 955 (5.50) | 2296 (13.22) |

B0=no childbirth before or in 2005, nor during the subsequent 3.75 years, B1=first childbirth in 2005 and no births during the subsequent 3.75 years, B1+=first childbirth in 2005 and at least one more birth during the subsequent 3.75 years.
